# Anomalous Motion Illusion Contributes to Visual Preference

**DOI:** 10.3389/fpsyg.2012.00528

**Published:** 2012-11-29

**Authors:** Jasmina Stevanov, Branka Spehar, Hiroshi Ashida, Akiyoshi Kitaoka

**Affiliations:** ^1^Faculty of Letters, Department of Psychology, Ritsumeikan UniversityKyoto, Japan; ^2^School of Psychology, University of New South WalesSydney, NSW, Australia; ^3^Department of Psychology, Graduate School of Letters, Kyoto UniversityKyoto, Japan

**Keywords:** motion illusion, esthetic preference, illusion magnitude, geometry of patterns

## Abstract

This study investigated the relationship between the magnitude of illusory motion in the variants of the “Rotating Snakes” pattern and the visual preference among such patterns. In Experiment 1 we manipulated the outer contour and the internal geometrical structure of the figure to test for corresponding modulations in the perceived illusion magnitude. The strength of illusory motion was estimated by the method of adjustment where the speed of a standard moving figure was matched to the speed of the perceived illusory motion in test figures. We observed modulation of the perceived strength of illusory motion congruent with our geometrical manipulations. In Experiment 2, we directly compared the magnitude of the perceived illusory motion and the preference for these patterns by a method of paired comparison. Images differing in illusion magnitude showed corresponding differences in the reported preference for these patterns. In addition, further analysis revealed that the geometry and lower level image characteristics also substantially contributed to the observed preference ratings. Together these results support the idea that presence of illusory effect and geometrical characteristics determine affective preference for images, as they may be regarded as more interesting, surprising, or fascinating.

## Introduction

While the investigations of artworks and other esthetically designed objects have dominated the area of experimental esthetics, many other visual experiences can, in addition, be considered as visually interesting, pleasant, and fascinating as well as emotionally evocative. Extending back over centuries, the intriguing properties of visual illusions in particular have attracted attention of ancient thinkers, philosophers, art impressionists, op artists, and contemporary artistic illusionists (Wade, 2005). People are generally fascinated or moved by various visual illusory effects, and it seems that such experiences are both perceptually and emotionally rewarding.

There has been a considerable amount of conceptual confusion in empirical esthetics regarding *a priori* definitions of esthetic preference, esthetic judgment, and esthetic experience. In the present study, we will use the term “visual preference” interchangeably with the term “esthetic preference.” McWhinnie’s (1968) definition of esthetic preference, which is widely accepted among researchers, refers to the degree with which people like a particular visual stimulus, how they rate “its” beauty or how much they prefer it to another. Since the word “esthetic” sometimes denote “artistic” meaning and sometimes denote “pleasantness” or “attractiveness,” we opted for more neutral term “visual preference.”

Noguchi (2003) studied the visual preference of several geometrical illusions: Oppel–Kundt grid and concentric circles, Helmholtz radials, the Delboeuf illusion, the Morinaga–Noguchi illusion, the Ehrenstein figure, and the Kanizsa–Noguchi square. His study revealed a strong correlation between affective and perceptual judgments, i.e., strong affective preference occurred with strong illusory effects.

In our recent study (Stevanov et al., 2012) we tried to cover a wider range of illusory patterns and measure esthetic and affective contribution of illusion to the preference of such visual patterns. We used geometrical illusions (e.g., the Luckiesh pattern), lightness illusions (e.g., the Anderson illusion, the enhanced Cornsweet effect), motion illusion (the Rotating snakes illusion), as well as other related illusory phenomena, such as ambiguous figures (e.g., the Necker cube, the Angel columns figure – ground reversible figure, the Frog-Horse figure) and impossible figures (e.g., Penrose’s triangle). Each of the illusory patterns was studied in its intact version as well as in modified version that was intended to produce a weakened strength of illusion (weak illusory variant). Our results were consistent with the same general pattern as observed by Noguchi: illusory patterns were esthetically preferred over their reduced-or-non-illusory counterparts. One exception to this pattern was the Rotating Snakes illusion (Kitaoka, 2008a,b), where, surprisingly, changes in the magnitude of motion illusion were unrelated to their esthetic preference. We speculated that the abundant richness and colorfulness of the figure pattern, present in both the weak and strong illusory variants, might have masked the differences between the illusory and non-illusory counterparts, compared to, arguably, “less colorful” remaining experimental stimuli. Another possible reason comes from certain limitations of the method used. Changes in illusion magnitude were introduced in a binary fashion: observers compared two figures, one of which had no illusory motion and the other which induced illusory motion. This method is particularly appropriate when only a few discrete interpretations exist, e.g., bi-stable ambiguous images, impossible figures, or figure-ground reversible images. In contrast, anomalous motion illusions have arguably more continuous illusion strength and may require more sensitive measures of reported esthetic preference.

Since anomalous motion illusions in general are relatively new as compared to others, it is worthwhile to look more closely how these illusory patterns affect preference. Across two experiments, the present study focused on parametric changes in the perceived magnitude of the Rotating Snakes illusion, and how these changes might be associated with the level of visual preference. To this aim, we need precise quantification of the illusion magnitudes to ensure that the differences are significant and that the set of stimuli reflects a certain gradient in perceived illusion strength. In Experiment 1, we introduced geometrical manipulations of the original illusory pattern and measured the illusion strength by the method of adjustment: the speed of the standard moving figure was matched to the speed of the perceived illusory motion in test figures. The expected modulation of the illusion strength was confirmed as geometry was manipulated, and in Experiment 2, we probed the relationship between the illusion magnitude and the visual preference. Experiment 2 used a set of stimuli chosen from the previous experiment and utilized a method of paired comparisons to establish concurrent and more comparable measures of the illusion magnitude and visual preference.

## Experiment 1

There are a variety of static images that induce illusory motion; some triggering illusory motion spontaneously while others require movement of the retinal image (Kitaoka and Ashida, 2007). Fraser and Wilcox (1979) designed a motion illusion with repeated sequences of a sawtooth luminance profile filling up the shape of a spiral. The “Rotating Snakes” illusion (Kitaoka, 2003), which could be considered an enhanced version of the Fraser–Wilcox illusion (Kitaoka and Ashida, 2003; Backus and Oruç, 2005; Kitaoka, 2007), enforces consistent direction of illusory motion, with subunits of stepwise luminance changes (black-dark gray-white-light gray) placed along the circumference of multiple concentric circles.

The basic pattern used in the current study is a simplified version of the “Rotating Snakes” and is classified as Optimized Fraser–Wilcox illusion type IIa (Kitaoka, 2006, 2007), which is hereinafter referred to as the “simplified Rotating Snakes” illusion. Previous studies identified many important properties that affect illusion magnitude (for review see Backus and Oruç, 2005; Conway et al., 2005), among which it was shown that contrast changes strongly affect perceived illusion strength. However, to fit the purpose of this study, we need simple manipulations that can modulate the illusion magnitude without changing the contrast that could greatly affect preference judgments regardless of illusion magnitude. Therefore, we changed the geometry of the pattern in the figures with minimum changes to the micropatterns. There is anecdotal evidence that the illusion is stronger when the color patches are arranged circularly, or possibly radially organized in comparison to the columnar and other arrangements (Fermüller et al., 2010). Thus the outer contour and the inner layout of micropatterns were independently deformed to be between square-columnar and circle-radial arrangement, and we tested whether a decrease in illusion magnitude is better predicted by internal area- or contour-related changes. In Experiment 1, we measured the magnitude of illusory motion by the method of adjustment where the speed of the standard moving figure was matched to the speed of the perceived illusory motion in test figures. Modulation of illusion magnitude was confirmed as the geometry was manipulated.

### Materials and methods

#### Participants

Twenty-nine students of psychology at the University of Belgrade (aged between 19 and 22) participated in the experiment. All participants signed informed consent for voluntary participation in exchange for the course credits. Participants had normal or corrected-to-normal vision. Before the experimental trials, we presented participants with green-purple version of the “simplified Rotating Snakes” illusion (Kitaoka, 2006, 2007) and all of the participants reported that they saw disks rotating in the expected directions.

#### Stimuli

**Figure 1:**
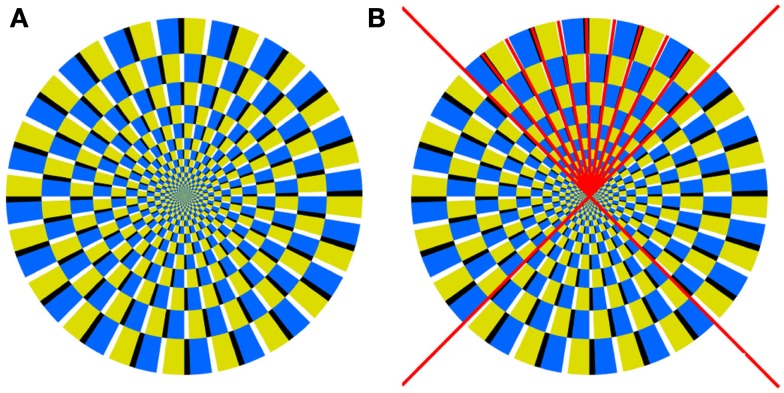
**(A)** The basic pattern used in Experiment 1. This pattern appears to rotate counterclockwise. **(B)** The auxiliary radii lines, shown in red in the top quadrant.

We used a “simplified Rotating Snakes” pattern that was introduced by Kitaoka (2006, 2007; Figure 1). Each subunit has areas of different luminance levels; a thin area of black or white (the darkest or the lightest) is flanked by thicker areas of yellow and blue.

For convenience, we draw auxiliary radii lines along which the color segments are placed (Figure 1B). In this way the pattern geometry can be defined as a function of the circle radius.

**Figure 2:**
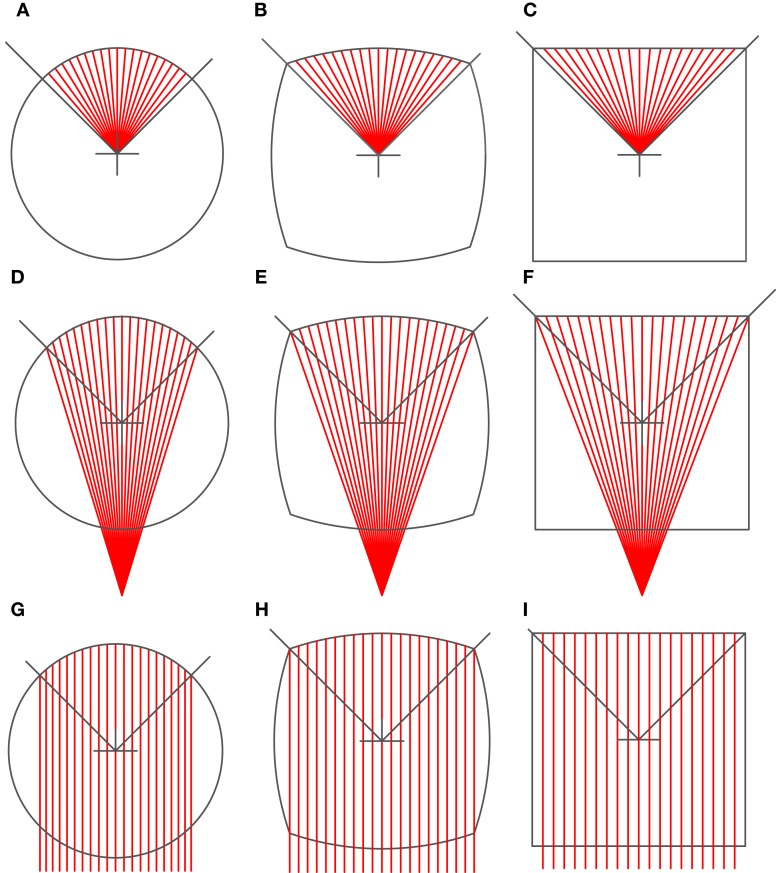
**Various Deformations of auxiliary radii lines. (A)** circle-radial, **(B)** circlesquare-radial, **(C)** square-radial, **(D)** circle-elliptic, **(E)** circlesquare-elliptic, **(F)** square-elliptic, **(G)** circle-paraboloidal, **(H)** circlesquare-paraboloidal, **(I)** square-paraboloidal.

**Figure 3:**
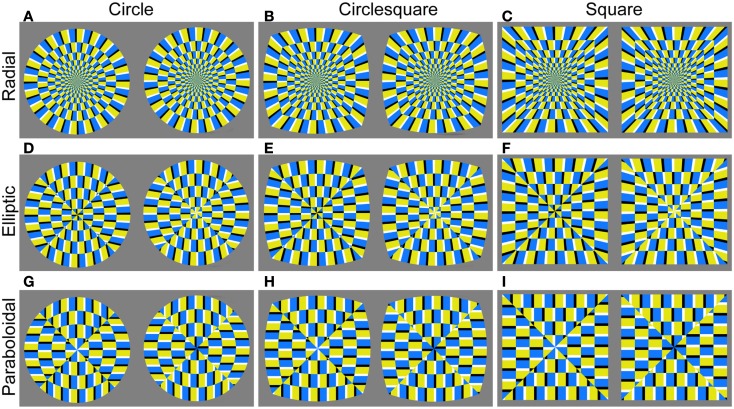
**The deformed figures that comprised the experimental set of stimuli (A–I)**. Reversed color orders in each pair of figures create illusory rotation in opposite direction. A left figure in each stimulus had counterclockwise rotation whilst the right one was rotating in clockwise direction.

If we move the center of the red auxiliary radii lines down (going from Figures 2A–I), the curvature of the bounding arc decreases. When the center approaches infinity, the auxiliary lines as well as the bounding contour line become straight (Figure 2I). Each quadrant was separately deformed along these auxiliary radii. Using this principle, the outer contour and the inner layout of the colored subunits were separately deformed to create test figures (Figures 3A–I). This allowed us to uncouple the contour from the enclosed area, and to test whether illusion magnitude could be better associated with changes in the appearance of the inner structure or those in the contour curvature respectively. In total there were three types of the *Contour* (*circle*, *circlesquare*, *square*) and three types of the *Inner layout* (we will call them *radial*, *elliptic*, *paraboloidal*). With those conditions combined, we created nine figures in total (Figure 3).

#### Procedure

The experiment was carried out in a dark room. Stimuli were generated with an Apple Mac Book Pro computer running Windows XP, and displayed on its 13′′ LCD display with the resolution of 1280 × 800 pixels, maximum luminance of 128 cd/m^2^, and the refresh rate of 60 Hz. Participants were seated 50 cm away from the display. We used the method of adjustment to estimate the speed of perceived illusion in each test figure. The illusion is stronger when seen in peripheral vision (Hisakata and Murakami, 2008). Therefore, two test figures were presented to the left and right. The height (radius) of all test figures was constant of 330 pixels (subtending 6.3° of visual angle horizontally and vertically).

The distance between their centers was 835 pixels (16.5°). The color orders in the two test figures were arranged differently, so that the left one appeared to rotate in counterclockwise direction and the right one appeared to rotate in clockwise direction.

**Figure 4:**
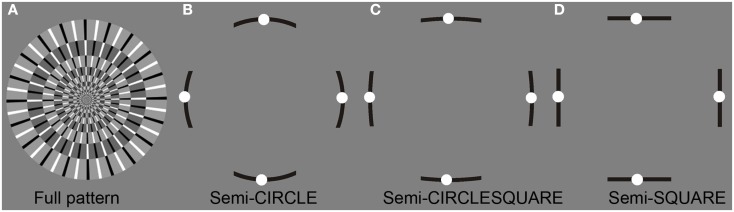
**Two types of the standard comparison figure: (A) the full pattern which resembled the pattern of test figures**. **(B–D)** The semi-contour figures with four white dots placed along each of the four contour segments. The contour shape of the semi-contour figure varied with the test figures.

A standard comparison figure was placed between the two test figures. We used two types of standard comparison figures (Figure 4): (1) the full pattern of disk image whose pattern resembled the pattern of test figures, and (2) the semi-contour line figure that consisted of four contour segments with four white dots placed along each segment. The full pattern always had a circle contour and radial layout, while the line segments in the semi-contour stimuli always had the same contour shape as the test figures. The whole image of the full pattern could be set in rotation, while only white dots of the semi-contour figure could be moved along the contour line. The reason for introducing the semi-contour figure was that the disk-like full-pattern figure would not match the shape or the inner layout of all test stimuli, while it did not seem appropriate to have figures with circlesquare and square contours rotating. Another reason for using the line segments was that several observers in our pilot study reported no or very weak illusory motion around the corners and along the quadrants boundaries in some test figures. Furthermore, the comparison figure moved at a constant angular speed, which may not be an appropriate measure of speed in circlesquare and square shaped test figures. Here semi-contour line figures worked as a compromise solution. Theoretically, the angular velocity and linear velocity of points in these contour segments would not deviate much across semi-circle, semi-circlesquare, and semi-square contour figures (Figure 4).

A custom-written software (in C#) was used to simultaneously generate stimuli and record speed estimates. The standard comparison figure could be set in motion and speeded up or down in desired direction using speed control buttons with arrows indicating the corresponding counterclockwise or clockwise directions. The speed unit was set in a range from 1 to 1000, where 1000 corresponded to 1 rad/s.

Participants viewed the stimuli binocularly without a chin-rest. They were instructed to observe the test figures freely for at least 5 s, without fixating any point on a screen, since hard fixation could weaken the illusion is intensified by eye movements (Murakami et al., 2006). After this observation, the task was to adjust the direction and the physical speed of the standard comparison figure to match the apparent direction and speed of the illusory motion in the test figures. A red arrow appeared randomly above the left or the right test figure to indicate which figure should be adjusted on that particular trial. In some figures participants may have perceived a transient motion, which decays rapidly. In such cases they were instructed to report the speed of the transient phase they captured. Only if the figure did not appear to rotate at all for the whole observation period (minimum 5 s), they could proceed to the next trial without moving the standard comparison figure. When judging the perceived speed of illusory motion in test figures, observers were told not to attend to only the outermost ring of the test figure, but to make an effort to observe the whole figure. A total 36 trials were randomly conducted for each participant. The whole session lasted between 20 and 30 min. Participants could take a rest in between the trials whenever they needed.

### Results and discussion

A four-way repeated measures analysis of variance was conducted in order to test for the specific effects of all independent variables specified in the design: (1) *Contour* (*Circle* Mean 4.64, SD 2.35; *Circlesquare* Mean 4.20, SD 2.16, *Square* Mean 3.48, SD 2.12), (2) *Inner layout* (*Radial layout* Mean 4.20, SD 2.15; *Elliptic layout* Mean 4.17, SD 2.10; *Paraboloidal layout* Mean 3.94, SD 2.29), (3) *Motion direction* (*Counterclockwise motion direction* Mean 4.03, SD 2.24; *Clockwise motion direction* Mean 4.18, SD 2.03), (4) *Standard comparison figure* (*Standard comparison figure of the full disk pattern* Mean 4.32, SD 2.02; *Semi-contour line figure* Mean 3.89, SD 2.24). This analysis revealed no significant main effect of the Motion direction [*F*(1,28) = 0.44, *p* = 0.51] or interactions with other independent variables (*p* > 0.11 for all). Further, we found no significant main effect of the Standard comparison figure [*F*(1,28) = 3.53, *p* = 0.07] or interactions with other independent variables (*p* > 0.17 for all). For simplicity, therefore, we collapsed the data across two motion directions and two types of standard comparison figure in further analysis of the Contour and Inner layout effects on perceived speed of illusory motion.

**Figure 5:**
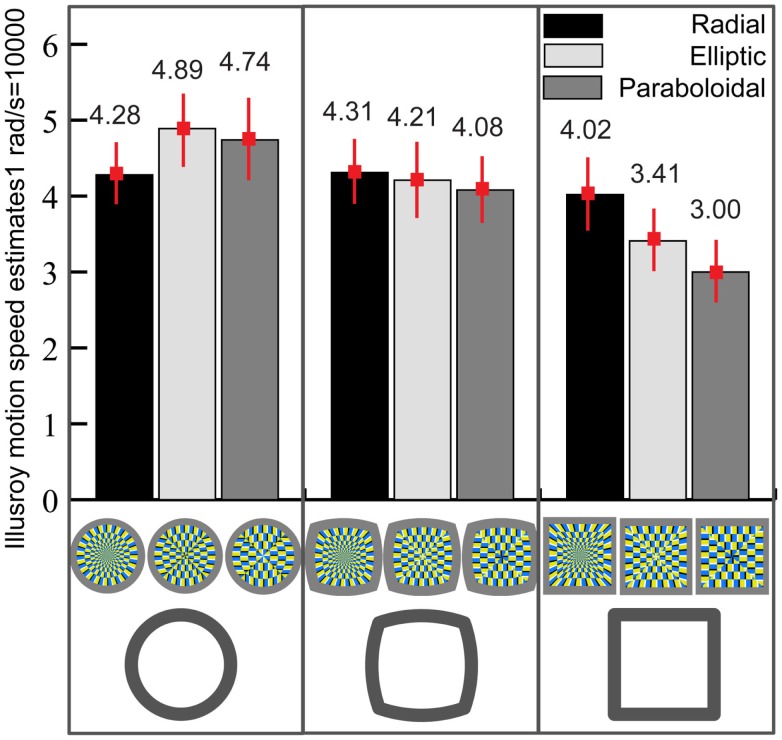
**The interaction between the contour and the layout**. Vertical bars denote ± standard errors. Speed unit was set in a range from 1 to 1000. Speed of 1000 corresponded to 1 rad/s.

Figure 5 shows the estimated magnitude of illusory motion for each condition. A two-way repeated measures analysis of variance revealed a significant main effect of *Contour* on the magnitude of illusory motion [*F*(2,56) = 9.21, *p* < 0.001]. Partial difference was significant between *Circle* and *Square* (*p* < 0.001) and between *Circlesquare* and *Square* (*p* = 0.03), but not between *Circle* and *Circlesquare* (*p* = 0.11). On the other hand, the effect of *Inner layout* was not significant [*F*(2,56) = 0.70, *p* = 0.50]. In addition, there was a significant interaction between *Contour* and *Inner Layout* [*F*(4,112) = 3.21, *p* = 0.01], suggesting that the effect of the inner layout may be different for each type of contour.

Second order partial difference between the square-radial and the square-paraboloidal figures was significant [*t*(28) = 1.14, *p* = 0.006], whereas the partial difference between the square-radial and the square-elliptic figures reached marginal significance (*p* = 0.056).

Similarly, partial differences revealed that the circular-radial figure yielded significantly lower speed estimates than the circular-elliptic one [*t*(28) = 2.19, *p* = 0.037], but not significantly lower than the circular-paraboloidal figure [*t*(28) = 1.14, *p* = 0.26]. This probably would explain why the interaction between the contour and the layout reached significance.

We have confirmed that systematic changes in illusion strength can be derived by the systematic changes in visual appearance of the test figures. The contour was central to illusion strength and the inner layout showed some different effects within different figure contours. Because the difference between Circle and Circlesquare was not clear, Circle and Square figures were selected as stimuli for the Experiment 2, which aimed at testing the relationship between the illusion magnitude and the visual preference.

## Experiment 2

In our previous study (Stevanov et al., 2012) we employed rating scaling method to map the difference between non-illusory and illusory stimuli on a number of implicit attributes such as interestingness, complexity, diversity, pleasantness, fascination, etc. In present study we sought to probe the relationship between the perceived illusion magnitude and visual preference more directly. The stimuli were selected on a basis of Experiment 1 and were assessed for both the perceived illusory motion magnitude and visual preference using a method of paired comparison. In this procedure, each image is paired with every other image in a set with all paired image combinations presented with equal frequency. The main advantage of this procedure is that throughout the experiment, the observers make comparisons (and or relative preference) between only two images at the time, thus minimizing both task complexity and memory demands (McManus et al., 2010).

### Materials and methods

#### Participants

Sixty students of psychology at the University of New South Wales (aged between 19 and 22) participated in the experiment: 30 participants did the magnitude task and 30 participants did the preference task. All had normal or corrected-to-normal vision and were naïve to the purpose of the experiment and to the stimuli. All participants reported that they could see illusory motion in one of the color variations of the simplified Rotating snakes illusion before proceeding to the main experimental task (Kitaoka, 2006, 2007). All participants signed informed consent for voluntary participation in exchange for the course credits.

#### Design

We employed between-participant design to establish the relationship between illusion magnitude and the visual preference. The between-subject design was chosen over within-subject design to avoid several practical and theoretical disadvantages. Firstly, there is a problem of carryover effects, where the first task (magnitude or preference task) would adversely influence the other, leading to a spurious correlation between the two measurements. This problem could not be resolved by counterbalancing the tasks, because there is a reasonable doubt that the same participant would have substantial difficulty in setting completely different response criteria for the judgments of the preference and the illusion strength. Another, more important, concern is that counterbalancing the tasks would also render a new confounding effect that is better to be avoided: it is well documented that there is a strong relationship between the familiarity and the preference, suggesting that familiarity increases the preference (Tomkins, 1962; see Cupchik and Gebotys, 1990; Consedine et al., 2004; Silvia, 2006; Sander and Scherer, 2009). Consequently, half of the participants would build up higher cumulative familiarity with the stimuli, leading to inequality of experimental conditions and offset between the preference functions of the two groups of participants.

#### Stimuli

**Figure 6:**
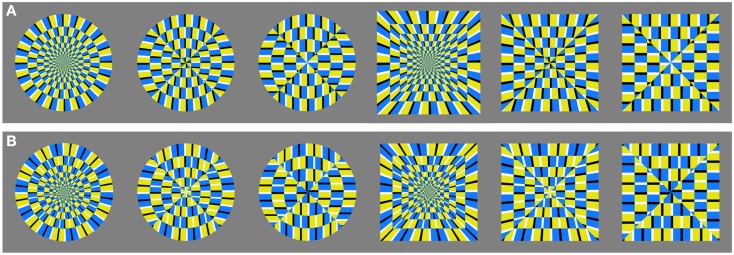
**The whole set of stimulus images: (A) the upper panel shows illusory figures, while the lower one (B) shows their non-illusory counterparts: they were matched in features like contour and inner layout, but were not supposed to evoke any illusory motion**.

We picked the circle- and square-contour test images from the first experiment, which significantly differed in illusion magnitude. Each test image had two figures side by side, with reversed color orders, so that the left one appeared to rotate in counterclockwise direction and the right one appeared to rotate in clockwise direction. In addition to these weak-to-strong illusory images, we created non-illusory figures in which the order of colors in adjacent subunits was reversed so that the overall motion signal was nulled. These non-illusory counterparts were introduced as control stimuli. They were matched in features like contour and inner layout of subunits, but did not evoke global illusory motion (Figure 6). Although they may produce some jittering motion, Kuriki et al. (2008) examined fMRI responses in motion sensitive areas of the human visual cortex (hMT+) and revealed significantly higher activity for the illusory figure in comparison with the “non-illusory” one that was created in the same way. Non-illusory test images also had two figures side by side. The color of the thin area (black-white) flanked by blue or yellow was reversed in two adjacent figures.

#### Procedure

Experimental conditions were similar to the first experiment. Stimuli were shown on a computer screen (19′′ CRT display with the resolution of 1280 × 800 pixels, maximum luminance of 250 cd/m^2^ and refresh rate of 60 Hz) in a darkened room. Participants were seated 70 cm away from the display. The height (radius) of all test figures was 330 pixels (subtending 6.3° of visual angle horizontally or vertically), as in the Experiment 1. Distance between the centers of the test figures was 400 pixels (7.4°). Visual stimuli were shown using Matlab and custom-written code in Psychtoolbox (Brainard, 1997; Pelli, 1997) running on Windows 98.

For the purpose of paired comparisons, 12 experimental stimuli were each paired with every other yielding a total of 66 experimental pairs or choice sets. Each of the paired sets was presented twice with the order of stimulus presentations counterbalanced; i.e., each experimental stimulus appeared equally often as the first as well as the second stimulus in a paired set. A two-interval variant of the paired comparison procedure was used such that in each paired choice set, experimental stimuli were shown one at a time and observers were requested to compare the stimuli presented in successive temporal intervals. The observers were able to go back and forth as many times as it was necessary for them to decide which one they preferred (*preference task*) or which one seemed to have greater illusion (*magnitude task*). Each experimental stimulus was presented 22 times in total: 11 times in the first temporal interval and 11 times in the second interval. In total, each observer made 132 choices between paired experimental stimuli.

In the magnitude task, observers were instructed to freely observe test figures without fixation so that the illusion would persist for the whole observation period. We gave no further instruction on how to base their judgments of the illusion magnitude.

In the preference task, the instruction to the observers stressed that there were “no right or wrong” answers, and that they should rely only on their subjective impressions. They were told to indicate which of the two images they prefer, i.e., which looks more pleasing or more attractive to them (McWhinnie, 1968; McManus et al., 2010). Observers were told to base their responses on their feeling about the images at that moment, without reflecting on their choices made on previous trials.

### Results and discussion

#### Correlation between illusion magnitude and preference

The visual preference and the illusion strength were quantified in terms of the proportion of times each image was chosen. If there are no systematic differences among stimuli, the relative frequency with which each image is chosen will not vary across images.

**Figure 7:**
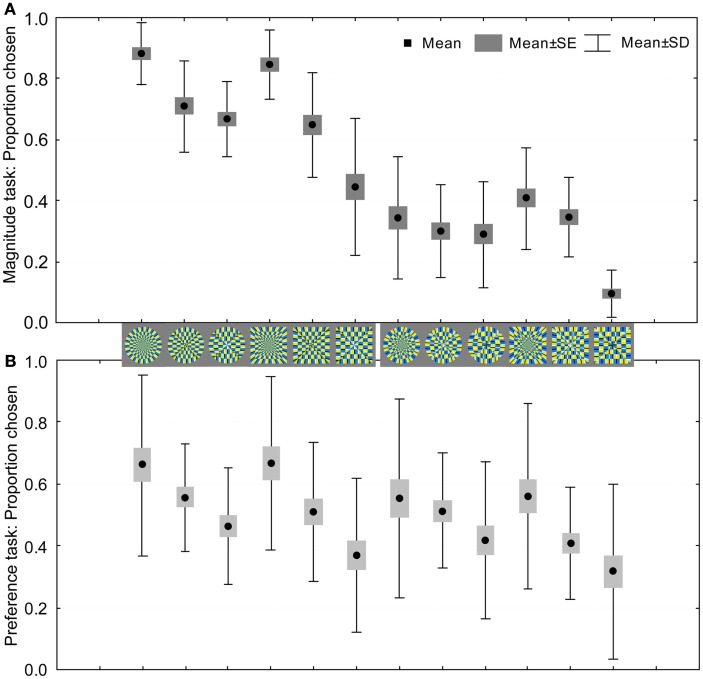
**Visual preference for weak-to-strong illusory and non-illusory images: average proportion by which the image was chosen among others as a function of illusion magnitude (A); visual preference of weak-to-strong illusory and non-illusory images quantified in terms of the proportion of times each image was chosen (B)**.

The illusion magnitude and preference data are plotted in Figure 7. The top panel shows that the circle-radial (leftmost) and the square-radial (fourth from the left) figures were ranked the highest for illusion magnitude in the set, followed by circle-elliptic, circle-paraboloidal, and then by square-elliptic and square-paraboloidal figures. It is also evident that the circular figures are ranked higher than the square figures, which agrees with the results of Experiment 1, suggesting that the circle contour promotes illusory motion. The non-illusory images were ranked the lowest, and there were no systematic differences among the non-illusory images. Similarly, the bottom panel depicts the preference results whose pattern closely follows the changes in inner layout, suggesting that the radial layout was better preferred over the elliptic or the paraboloidal one.

We obtained a high positive correlation between the preference judgments and perceived illusion magnitude [Pearson’s correlation *r*(10) = 0.76, *p* = 0.004]. When we omit the non-illusory counterparts, correlation between magnitude and preference for the illusory images was even higher [*r*(4) = 0.97, *p* = 0.001]. Central feature of these results is a high degree of similarity in patterns of the preference and magnitude responses.

#### Individual differences and *Q*-mode factorial analysis

Although the main purpose of this study was not to describe individual differences, it is, however, useful to understand what aspects of images interact in expression of their preferences. The Pearson’s pairwise correlation between the preference estimates of every pair of participants averaged 0.11 (SD = 0.48), whereas the pairwise correlations of the illusion magnitudes averaged 0.70 (SD = 0.23). Relatively low agreement across participants for preference judgments might imply that participants based their preference responses on different principles or aspects of the images.

In order to investigate further what governed preference judgments of different participants, we applied the *Q*-mode factorial analysis [a method proposed by McManus (1980) and McManus et al. (2010)]. For this analysis, we use correlations between the preference estimates of every pair of participants, as opposed to conventional factor analysis that uses correlations between the stimuli. Therefore, this analysis extracts factors that can reveal the underlying criteria which governed their preference judgments, and the factor loadings for each participant on extracted factors will show how each participant puts more or less weight to each criterion.

**Figure 8:**
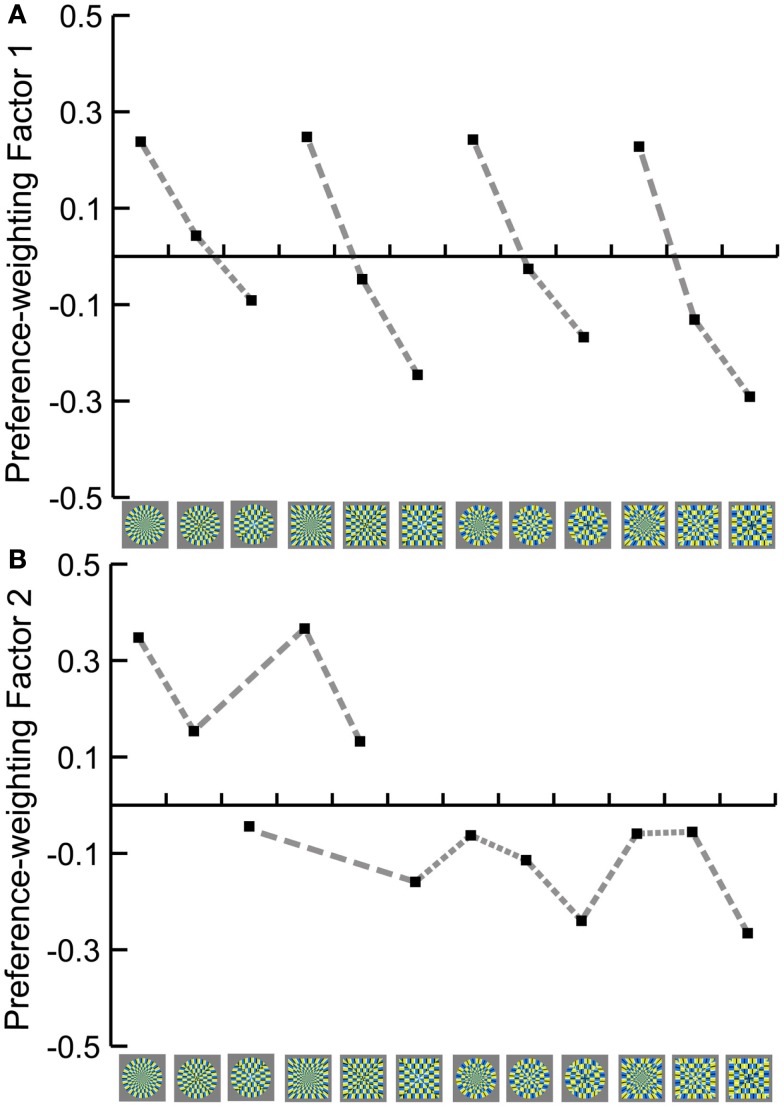
**Summary preference functions for (A) Factor 1 (radial, elliptic, and paraboloidal layout) and (B) Factor 2 (illusion)**. The functions were calculated from the preference functions of all 30 participants, weighted by their loadings on the *Q*-mode factors, and then standardized.

We applied factor analysis with PCA extraction method followed by a Varimax rotation to the 30 × 30 correlation matrix. The Kaiser criterion suggested four factors with eigenvalues of 17.94, 6.26, 3.4, and 1.3, whereas the scree-slope analysis revealed two factors above the general “scree.” We therefore interpreted only the first two factors. The first factor accounted for 59.81% and the second factor for 20.88% of the total variance, summing up to 80.69% of the total variance explained by the first two extracted factors. In order to reify these two main factors, the participants’ preference matrix was then multiplied by the factor loadings of each subject on each of the two main factors. The resulting weighted matrices were summed and the totals were standardized so that the absolute total was equal to 2 (McManus, 1980). These standardized totals were then plotted against the stimulus set (Figure 8). This is a convenient way to single out and *a posteriori* interpret the criteria upon which the preference judgments were made. Naturally, its explanatory power is not the same as of the *a priori* specified criteria. It is, however, one of the possible statistical methods to underpin the structure of the preference.

In order to see if either of the two factors could be associated with the perceived illusion magnitude, we tested correlations between these weighted totals and the standardized Magnitude responses (derived from Figure 7). We obtained high positive correlation with the Preference responses weighted by the Factor 2: *r*(10) = 0.92, *p* < 0.001, whilst the correlation with the Preference responses weighted by the Factor 1 was not significant: *r*(10) = 0.55, *p* = 0.062, indicating that the Factor 2 mostly reflects the illusion magnitude. Since Preference responses weighted by Factor 1 and Factor 2 are correlated [*r*(10) = 0.70, *p* < 0.05), additionally, we calculated partial correlations between the Magnitude responses and the weighted preference responses alternately controlled for the effect of the Factor 1 or the Factor 2; high correlation between the Magnitude responses and the Preference responses weighted by the Factor 2 (adjusted for the effect of Factor 1) was confirmed: *r*(9) = 0.89, *t*(9) = 5.99, *p* < 0.001, whereas correlation between the Magnitude responses and the Preference responses weighted by the Factor 1 (adjusted for the effect of Factor 2) was not significant: *r*(9) = −0.31, *t*(9) = −0.99, *p* = 0.35, confirming that Factor 2 but not much of Factor 1 is related to the Magnitude responses.

**Figure 9:**
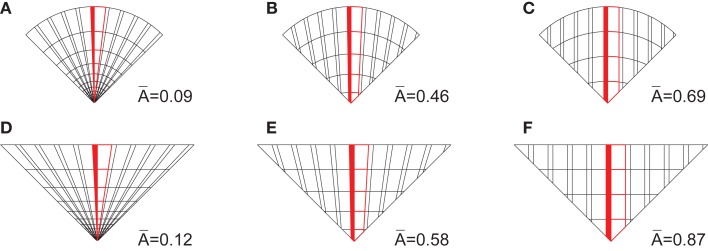
**Average segment size along the middle (in red) radius in one quadrant of the (A) circle-radial figures, (B) circle-elliptic figures, (C) circle-paraboloidal figures, (D) square-radial figures, (E) square-elliptic figures, and (F) square-paraboloidal figures**. The area of trapezoidal segments depends on the lengths of each base and the height, which is expressed as a function of the radius. The radius length = 5.

Interpretation of the Factor 1 is less straightforward. Whereas Factor 1 could be related to the inner layout and the shape of the figures, as suggested in Figure 8A, it could also reflect the differences in image features such as spatial frequency components. As one of the measures that could reflect changes in geometry of figures, we took the average size of the yellow/blue color segments, which extend along the middle vertical or horizontal radius equal in all figures. The segment size is defined as a function of the total area and the number of segments, which differs across the radial, elliptic, and paraboloidal inner layout (Figure 9).

Partial correlations revealed that the Segment size highly correlates with the Preference responses weighted by the Factor 1 when adjusted for the Factor 2: *r*(9) = −0.98, *t*(9) = −15.06, *p* < 0.000, whereas the Preference responses weighted by the Factor 2 do not correlate with the Segment size when adjusted for the Factor 1: *r*(9) = 0.37, *t*(9) = 1.21, *p* = 0.26.

**Figure 10:**
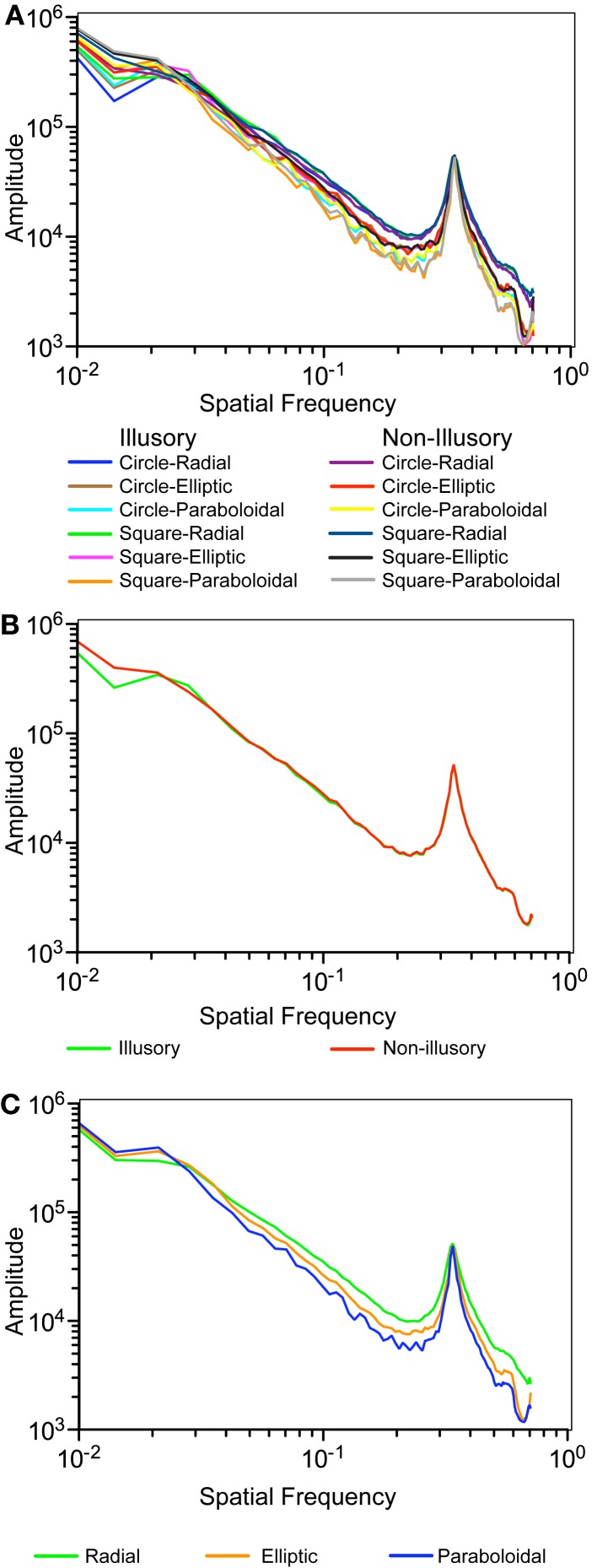
**Amplitude spectra of Spatial Frequency: (A) individual stimuli (B) averaged for the Illusory vs. Non-illusory stimuli (C) averaged for the three types of the Inner Layout**.

Naturally, the size of the segments is related to the spatial frequency characteristics of these patterns. Relatively speaking, as the size of the component segments decrease, there should be more energy associated with high spatial frequency. Therefore, the slopes of the fitted linear functions to the log amplitude spectra for each of the stimuli (Figures 10A–C) were expected to be correlated with the weighted Preference responses. As predicted, partial correlation of the slopes with the Preference responses weighted by the Factor 1 (adjusted for the Factor 2) was high: *r*(9) = 0.92, *t*(9) = 6.86, *p* < 0.000, whereas the partial correlation with the Preference responses weighted by the Factor 2 (adjusted for the Factor 1) was not significant: *r*(9) = −0.158, *t*(9) = −0.48, *p* = 0.64.

These results suggest that the geometry changes of the stimuli (described either in space or frequency domain) could explain the nature of the Factor 1, while theoretically other unsubstantiated features of the figures may be involved as well. While the explanation of the Factor 1 is not of our primary importance to the purpose of this study, essential is that the geometry features of the stimuli are not much reflected in the Factor 2. Therefore, we conclude that the illusion magnitude could be attributed to Factor 2.

**Figure 11:**
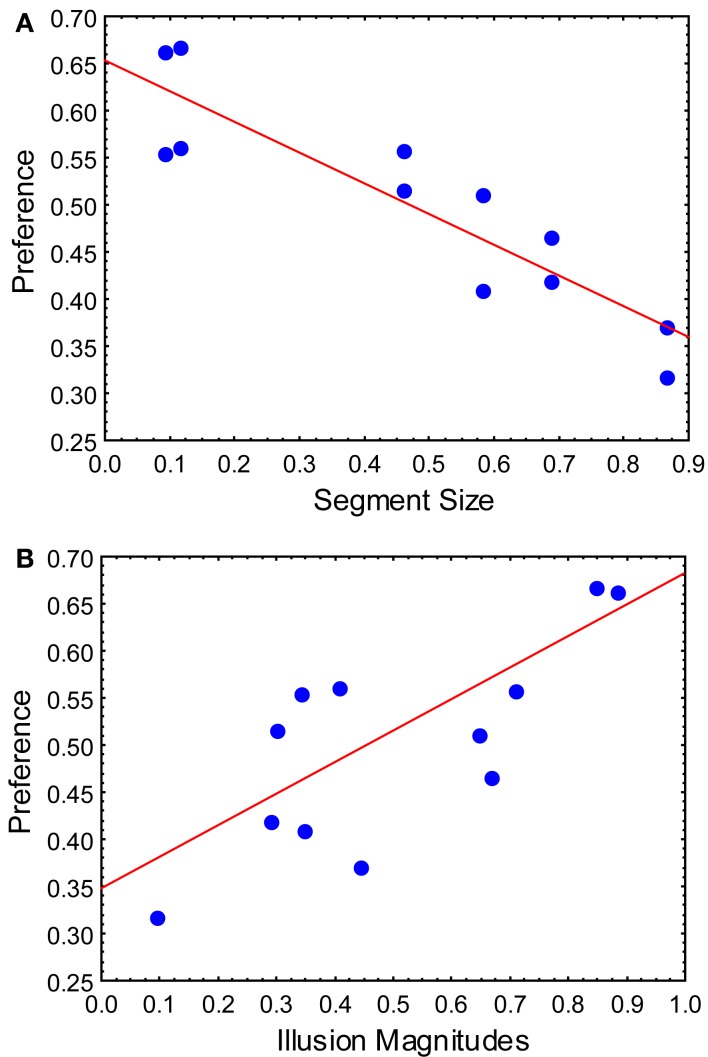
**Scatterplots of the regression analysis: a criterion variable was the Preference and predictors were (A) the Segment size and (B) the Illusion Magnitudes**.

Now that we have defined major determinants of the visual preference, we applied a multiple regression analysis to investigate which of these suggested determinants are more important than the others. The raw Illusion Magnitude responses from Figure 7, the Segment size and the Spatial Frequency values were taken as predictors in a regression model with the raw Preference responses (derived from Figure 7) as the criterion variable. We applied backward stepwise multiple regression, which begins with an examination of the combined effect of all predictors on the criterion variable. Starting with the weakest predictor, variables were excluded from the model and a new analysis was performed. Resulting coefficients refer to the degree to which each predictor contributes to predicting the dependent variable. In the analysis, the Spatial Frequency was a predictor that was first excluded: *t*(8) = −1.242, *p* = 0.25. The Illusion Magnitude responses were significant predictor of the Preference responses: β = 0.43, *t*(9) = 5.86, *p* < 0.001, as well as the Segment size: β = −0.70, *t*(9) = −9.45, *p* < 0.001. For the two remained predictors, we used Fisher’s *Z* score for comparing two correlated partial correlations (Meng et al., 1992). We tested the difference between the partial correlation of the Magnitude responses [*r*(9) = 0.89] and of the Segment size [*r*(9) = −0.95]. Fisher’s *Z* score was −0.94, two-tailed *p* = 0.35, suggesting no significant difference between the two predictors. In summary, both the Segment size and the Illusion Magnitude responses are good predictors of the preference responses [*R*^2^ = 0.96, *F*(2,9) = 110.41, *p* < 0.001; Figure 11].

## General Discussion

The general question posed in this study was “What is the relationship between the illusion magnitude and esthetic preference”? Noguchi (2003) proposed that affective preference increases as the illusion becomes stronger and the results of this study agree well with that notion. We compared differences in illusion magnitude with those in preference level of the “simplified Rotating Snakes” pattern, in which illusory motion is triggered spontaneously and persists. In Experiment 1 we tried to establish a way to parameterize the illusion magnitude without changing the basic attributes such as contrast that could greatly affect preference regardless of the illusion strength. A simple geometrical manipulation was used to deform the outer contour line and the inner layout of the micropatterns (segments ordered by luminance black-blue-white-yellow) to be between the square-columnar and the circle-radial arrangements. Speed estimates for these deformed figures were obtained and confirmed that the illusion was parametrically modulated as the geometry was manipulated. The results suggested that the curvature of the contour line is central to the perceived strength of illusion.

However, it was also shown that the inner layout had a different effect on illusion magnitude within the circle-shaped figures. It is counterintuitive that the elliptic layout of subunits enclosed by circular contour, gave rise to the strongest illusion, rather than the one with the radial layout, in Experiment 1. We could speculate that the two components involved in this illusion might have influenced the result, one being a transient phase that could be triggered by microsaccades or blinks (Otero-Millan et al., 2012) and the other being a sustained effect that could be maintained by fixational eye movement (Murakami et al., 2006). These two phases have not been clearly distinguished so far. The transient phase may look faster than the sustained phase even in the radial-circular figures, but when the sustained phase persisted, participants likely made adjustment on the basis of this phase. As a result, it is possible that participants may depend more on the transient phase with the circle-elliptic figure but more on the sustained phase with the circle-radial figure, leading to apparent superiority of the circle-elliptic figure. This reversal only occurs with the circular contour since the sustained phase appears particularly persistent in the circle-radial figure. In Experiment 2, the circular-radial figure was actually ranked the highest in the set, suggesting that it elicits the strongest motion illusion of all tested figures, which is more consistent with a subjective validation we had prior to the experiments. Validity of this interpretation needs further empirical tests. Nevertheless, it raises an interesting question for a future research: Is it possible to isolate the transient and sustained phase in the illusory motion of the “Rotating Snakes” patterns only by changing the geometry of the figures?

In Experiment 2 we obtained preference and magnitude judgments for both weak-to-strong illusory patterns and their non-illusory counterparts. High correlation was obtained between preference and magnitude responses, suggesting that illusion magnitude plays a substantial role in visual preference.

Further, the *Q*-mode factor analysis on the participant-pairwise-correlations was utilized as explorative technique to define underlying criteria of the individual’s preference judgments. Two major factors were extracted that account for 80% of the variance in participants’ responses. The first factor, which highly correlates with geometry changes in visual appearance of the figures and their spatial frequency distribution, explains 60% of the individual differences in preference judgments, whereas 20% of the variance could be assigned to illusion magnitude. Note that this does not mean that contribution of illusion magnitude in each preference judgment is only 20%. Instead, these results implicate that the preference for visual appearance and spatial frequency varies more across participants than the preference for the strength of the illusion, suggesting that preference for figures with stronger illusion is rather straightforward.

On the other hand, multiple regression analysis revealed that the geometry changes and the illusion magnitude are both good predictors of the preference responses, implying that they are both important determinants of the visual preference. The *Q*-mode analysis is useful in suggesting *a posteriori* what are the determinants of the preference, without *a priori* criteria specified by researchers. Joint efforts of the *Q*-mode factor analysis and multiple regression analysis imply that the geometry changes and illusion strength are the two major and equally important determinants of the visual preference for the Rotating Snakes illusion.

Relying on Berlyne’s proposition (Berlyne, 1974) of collative variables (i.e., novelty, complexity, surprisingness, incongruity), we could speculate that stronger illusion increases our judgments of novelty, surprisingness, or interestingness and therefore it increases preference. There is also a possibility that increase in preference could be due to increase in the speed of the perceived motion and not illusion itself. It is an open question, but given that the illusory motion is processed at an early stage of motion detection in V1 (e.g., Conway et al., 2005; Ashida et al., 2012), distinction of real and illusory motion may not be crucial in terms of preference.

The implications of the current results could be important for future related research, where the same method of paired comparisons could be used to assess the preference of other illusory effects which have continuous illusion strength like the anomalous motion illusions. Our previous study (Stevanov et al., 2012) employed the rating scale method for assessing multidimensional “subjective evaluative meanings.” This method was suitable for the purpose of the study, since we covered a wider range of different illusory effects being assessed on numerous affective and cognitive dimensions interacting in esthetic experience. Nevertheless, mapping a large number of stimuli on several dimensions of subjective judgments may impose certain cognitive complexity and raises concern that participants may maintain separate mappings to separate rating scales, whereas paired comparison does not require memory of previous stimuli, or anticipation of the subsequent ones. Therefore paired comparison method may be useful in comparing diverse stimuli when we aim to have them mapped onto a single dimension (preference only), minimizing the cognitive complexity of the task (McManus et al., 2010).

The limitation of the results is that they infer correlation and not causality necessary for clarifying the underlying mechanism of the illusion-preference co-occurrence. However, there have been only few studies which addressed the correlation between the illusion magnitude and the esthetic preference. This study is among the first attempts to substantiate contribution of the illusion strength from the other determinants of the preference for illusion figures.

## Conflict of Interest Statement

The authors declare that the research was conducted in the absence of any commercial or financial relationships that could be construed as a potential conflict of interest.
